# Immune Checkpoint Inhibitor-Induced Diabetic Ketoacidosis: A Report of Four Cases and Literature Review

**DOI:** 10.3389/fendo.2020.00014

**Published:** 2020-01-28

**Authors:** A Ram Hong, Jee Hee Yoon, Hee Kyung Kim, Ho-Cheol Kang

**Affiliations:** Department of Internal Medicine, Chonnam National University Medical School, Gwangju, South Korea

**Keywords:** type 1 diabetes mellitus, diabetic ketoacidosis, immune checkpoint inhibitors, autoimmune, PD-1, PD-L1

## Abstract

Immune checkpoint inhibitors (ICIs) have been widely used in the treatment of various types of cancers worldwide. Although ICI-related autoimmune diabetes is a rare complication, it can be associated with a life-threatening condition, diabetic ketoacidosis (DKA). Here, we report the cases of four patients who presented with ICI-induced DKA in a tertiary center in Korea. Three patients were newly diagnosed with type 1 diabetes, and one patient was known to have a history of type 2 diabetes. All DKA cases were due to programmed cell death protein-1 (PD-1) or its ligand inhibitors (PD-L1). The mean age of the patients was 71.5 years, and the mean time for diagnosing the onset of DKA after starting ICIs was 15.8 weeks. Glutamic acid decarboxylase antibodies were positive in one patient (25%) who already had been treated with type 2 diabetes. All four patients showed improved antitumor responses after ICI therapy and are currently receiving insulin treatment for glycemic control, regardless of their continuation of ICIs. As there have been no practically available predictive biomarkers for the diagnosis of DKA or type 1 diabetes thus far, close monitoring of blood glucose levels is required in all patients receiving ICIs.

## Introduction

Immune checkpoint inhibitors (ICI) have emerged as a breakthrough in the treatment of advanced stage cancers, including non-small cell lung cancer, melanoma, renal cancer, head and neck cancer, and urothelial cancers (1). ICIs modulate an inhibitory immune response by blocking cytotoxic T-lymphocyte antigen-4 (CTLA-4), programmed cell death protein-1 (PD-1), or its ligand (PD-L1) (2). Despite their enormous benefits in anti-tumor efficacy, immune checkpoint blockades are associated with several immune-related adverse events (irAEs), including endocrinopathies (3). Endocrine irAEs are observed in 4–30% of patients (4). Although thyroid dysfunction and hypophysitis are the most prevalent endocrine irAE, type 1 diabetes is rare (<1.0%), but can be associated with a potentially life-threatening condition, diabetic ketoacidosis (DKA) (4, 5).

Recently, several reported cases and meta-analyses have been published regarding ICI-induced type 1 diabetes or DKA, and the most recent systemic review demonstrated ~90 cases worldwide (6–8). The majority of autoimmune diabetes were induced by PD-1 or PD-L1 blockades (9–11). This could be attributed to the different mechanisms for immune modulation against pancreatic islets between PD-1/PD-L1 and CTLA-4 inhibitors, and also the increased use of PD-1/PD-L1 inhibitors in clinical practice. There have yet been no reported ICI-induced type 1 diabetes in Korea.

Here, we describe four patients presenting DKA after ICI therapy in real-world practice to improve our knowledge of ICI-related type 1 diabetes, particularly in endocrine perspectives.

## Methods

This was a retrospective study conducted in Chonnam National University Hwasun Hospital. We retrieved all cancer patients receiving ICI therapy including CTLA-4 inhibitors [ipilimumab [Yervoy®]], PD-1 inhibitors [pembrolizumab [Keytruda®] and nivolumab [Opdivo®]], and PD-L1 inhibitors [atezolizumab [Tecentriq®] and durvalumab [Imfinzi®]] between April 2016 and August 2019. We excluded the patients who received ICIs through clinical trials in the analysis. Of the 587 patients assessed, four patients (0.7%) presented DKA during treatment. We collected their clinical and biochemical data by reviewing the medical records.

HbA1c level was determined using high-performance liquid chromatography (SST; Becton, Dickinson and Company, Franklin Lakes, NJ, USA). Fasting C-peptide was measured by immunoradiometric assay (SST; Becton, Dickinson and Company, Franklin Lakes, NJ, USA). Glutamic acid decarboxylase (GAD) antibody was measured by immunoradiometric assay (SST; Becton, Dickinson and Company, Franklin Lakes, NJ, USA) and insulin autoantibody (IAA) was measured by enzyme immunoassay (SST; Becton, Dickinson and Company, Franklin Lakes, NJ, USA).

## Description of The Cases

Clinical characteristics of patients are summarized in Table 1. The mean age of the patients was 71.5 years (range 65–78 years) and 50% of them were male. ICIs were administered for various cancer types—lung cancer, urothelial cancer, melanoma, and biliary tract cancer. Three patients were treated with PD-1 inhibitor, pembrolizumab, and one patient was treated with PD-L1 inhibitor, atezolizumab. From the four patients, three were newly diagnosed with type 1 diabetes, while one patient already had type 2 diabetes. The mean duration of the onset of DKA after starting ICI was 15.8 weeks (range 4–17 weeks). The mean HbA1c was 9.4% (range 5.8–11.4%). There appeared to be no correlation between HbA1c and the time of onset to DKA after ICI therapy with newly diagnosed type 1 diabetes. Serum C-peptide levels, which is an indicator for β-cell function, were significantly reduced in all patients. We did not observe other endocrine dysfunctions affecting the thyroid and adrenal glands at with DKA, however, one patient developed adrenal insufficiency 2 months after DKA.

**Table 1:** Characteristics of reported patients with ICI-associated DKA.

**Case No**.	**1**	**2**	**3**	**4**
Age (year)	76	67	78	65
Sex	Male	Male	Female	Female
Underlying cancer	Lung cancer	Urothelial cancer	Melanoma	Biliary cancer
Type of ICI	Pembrolizumab	Atezolizumab	Pembrolizumab	Pembrolizumab
Previous history of diabetes	No	No	Yes	No
HbA1c (4.4–6.4%)	10.4	9.8	11.4	5.8
C-peptide (0.3–3.8 ng/mL)	0.01	0.01	0.01	0.1
Glucose (mg/dL)	493	530	494	511
Serum pH	7.165	7.166	7.157	7.085
Serum osmolarity (280–295 mOsm/Kg)	307	341	306	318
Serum bicarbonate (mmol/L)	7.3	13.2	14.0	6.5
Urine ketone	++++	+++	++	++++
Time to diagnosis after starting ICI				
Number of doses	3	9	1	7
Onset in weeks	11	27	4	21
β-cell autoantibodies				
GAD Ab	Negative	Negative	Positive	Negative
IAA	Negative	-	Negative	Negative
Other endocrinopathies				
Thyroid dysfunction	No	No	No	No
Adrenal insufficiency	Yes	No	No	No
Tumor response	Partial response	Stable disease	Complete response	Partial response
ICI therapy after DKA	Stop	Continued	Stop	Stop

*ICI, immune checkpoint inhibitors; DKA, diabetic ketoacidosis; GAD Ab, glutamic acid decarboxylase antibody; IAA, insulin autoantibody*.

**Figure 1:**
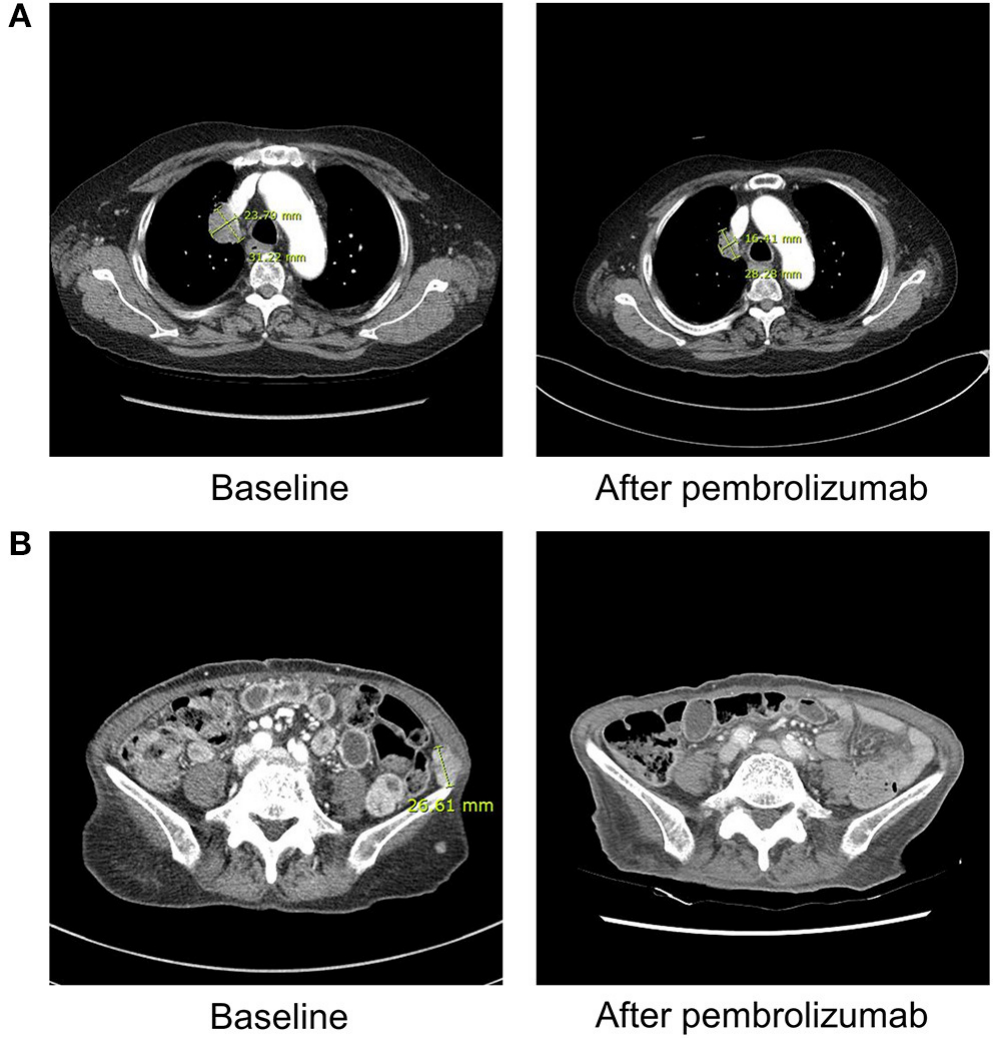
Radiographic findings obtained before and after ICI therapy in patient 1 and 3. **(A)** Patient 1 (with lung adenocarcinoma) shows markedly decreased size of right upper lung mass after three cycles of pembrolizumab on chest computed tomography. **(B)** Patient 3 (with malignant melanoma) demonstrates near complete remission of multiple abdominopelvic wall metastases after 1 cycle of pembrolizumab on abdominal computed tomography.

Although their tumor responses were considered as complete or partial response after ICI therapy, three patients stopped the treatment after the occurrence of DKA (Figure 1). Only one patient remained stable and continued ICI therapy with PD-L1 inhibitor (atezolizumab). Regardless of the continuation of ICIs, all patients continue to receive insulin therapy for their glycemic control.

### Case 1

A 76-year-old male patient presented with general weakness that started a week ago. He had been treated with pembrolizumab for lung cancer for 11 weeks (3 cycles) before the occurrence of DKA. He did not have a previous history of diabetes. Initial serum glucose level was 493 mg/dL, pH was 7.165, osmolarity was 307 mOsm/kg, bicarbonate was 7.3 mmol/L, and urine ketone was 4+. HbA1c level was 10.4% and C-peptide was 0.01 ng/mL. He did not have IAA or GAD antibodies. Despite a partial response to pembrolizumab, he stopped the treatment because of his poor general condition and new onset adrenal insufficiency.

### Case 2

A 67-year-old male patient presented with general weakness and polydipsia that started a month ago. He started atezolizumab for urothelial cancer 27 weeks (9 cycles) ago. He was not a diabetic patient. Initial laboratory findings showed a serum glucose level of 530 mg/dL, pH of 7.166, osmolarity of 341 mOsm/Kg, bicarbonate of 13.2 mmol/L, and urine ketone of 3+. HbA1c level was 9.8% and C-peptide was 0.01 ng/mL. GAD antibodies were not detected in the patient, and IAAs were not checked. No other endocrine dysfunctions were observed in the patient. He continues atezolizumab having a stable disease status under insulin injection therapy.

### Case 3

A 78-year-old female patient visited the emergency room due to hyperglycemia and general weakness. She started pembrolizumab 4 weeks ago (1 cycle) for melanoma. She had a 9-years history of type 2 diabetes and had been treated with triple oral anti-diabetic drugs. Her HbA1c level was 8.0% before starting pembrolizumab, and increased to 11.4% when DKA was detected. Initial serum glucose level was 494 mg/dL, pH was 7.157, osmolarity was 306 mOsm/Kg, bicarbonate was 14.0 mmol/L, and urine ketone was 2+. Serum C-peptide level was 0.01 ng/mL. Interestingly, she was tested positive for the presence of β-cell autoantibodies, GAD antibodies. Thyroid dysfunction or adrenal insufficiency was not observed. Despite the intensive insulin therapy, her glucose levels were not well-controlled. She even received high doses of steroid due to ICI-induced encephalitis 4 weeks after DKA. Although her cancer status showed nearly complete remission, she stopped the pembrolizumab due to poor general condition.

### Case 4

A 65-years old female patient complained of nausea and vomiting. She had been treated with pembrolizumab for 21 weeks due to biliary cancer. She did not have a history of diabetes. Initial laboratory assessment showed a serum glucose of 511 mg/dL, pH of 7.085, osmolarity of 318 mOsm/Kg, bicarbonate of 6.5 mmol/L, and urine ketone of 4+. GAD antibodies or IAAs were not detected in the patient. She did not show other endocrine dysfunctions. Although her glucose levels were well-controlled with insulin therapy, she discontinued the pembrolizumab after DKA because of the patient's demand.

## Discussion

To the best of our knowledge, these four cases are the first report regarding ICI-induced DKA or type 1 diabetes in Korea. In the current study, all DKA cases were induced by PD-1/PD-L1 inhibitors, mostly PD-1 inhibitor, pembrolizumab. The observed incidences of DKA was 0.7% (4/587), which is similar to previous reports (~1.0%) (5). There was no preponderance in cancer types related to autoimmune diabetes.

PD-1 is a receptor expressed on T cells that can be activated by its ligand, PD-L1. PD-1 and PD-L1 binding produces an inhibitory signal that regulates T-cell activation, leading to the suppression of immune system. PD-1 is also expressed on pancreatic islet cells. When PD-1 or PD-L1 blockade such as pembrolizumab is administrated, PD-L1 molecules of the pancreatic β-cells cannot bind to PD-1 on autoreactive T cells. Then, stimulated autoreactive T cells can survive and destroy the β-cells, leading to the development of diabetes (8).

The characteristics of our patients were generally similar to the previously reported ones, but different in several aspects. Our patients were older than previous ones with a mean age of 61 years and there was no gender differentiation reported for these patients. Previous studies showed that more than 70% of the new-onset type 1 diabetes initially presented DKA (6, 8). In our study, all patients with newly diagnosed type 1 diabetes were detected for the development of DKA. The mean duration of DKA onset after the start of ICIs was 15.8 weeks, which was relatively longer than previously reported. A recent meta-analysis reported the median duration of diabetes with PD-1/PD-L1 inhibitors as 49 days (range 5–448 days) and about 70% of the patients developed new-onset type 1 diabetes within 3 months (7). The time to the diagnosis of type 1 diabetes after initiating ICI therapy was not associated with the level of HbA1c, which implies the acute onset of hyperglycemia. The mean HbA1c levels were higher in our study compared to previous studies (9.4 vs. 7.5%), which was still higher when analyzed in only new-onset diabetes (8.7%) (6).

Previous results showed that the patients who had autoantibodies against β-cell, including GAD antibodies, IAAs, insulinoma antigen-2 antibodies, and islet cell antibodies, more likely presented DKA and within shorter time intervals from ICI therapy (12, 13). The reported prevalence of autoantibody positive rate was approximately 50% (6). Whereas, we observed only one case (25%) with autoantibodies in the current study. Remarkably, GAD antibodies were observed in the one patient who already had a history of type 2 diabetes, and she developed DKA after 1 cycle of pembrolizumab. However, it is unclear whether the GAD antibodies preceded the start of pembrolizumab or developed after receiving therapy. The possibility of coexisting latent-onset diabetes in adults is also not excluded. Although we examined only GAD antibodies and IAA, the prevalence of autoantibodies seems to be lower than those from previous reports considering the predominance of GAD antibodies in type 1 diabetes. Regardless of the presence of autoantibodies, serum C-peptide levels were very low, which is in line with previous results.

In the current study, we did not perform human leukocyte antigen (HLA) genotyping in the presented cases. In addition to autoantibodies, certain HLA genotypes are known to predispose significant risk for the development of type 1 diabetes including DR3-DQ2 and DR4-DQ8 haplotypes (14). Approximately 61% of patients with ICI-induced diabetes were associated with increased susceptibility for either type 1 diabetes or fulminant diabetes, mostly DR4 (6, 15). However, since HLA genotypes can only partially explain some individuals with a higher risk of diabetes, it is difficult to strongly suggest that HLA genotyping is performed in all patients who start ICI therapy.

The development of irAE, including cutaneous adverse events, has been associated with a better anti-tumor efficacy with an improved response rate and longer survival (16, 17). There is limited data regarding the relationships between the development of type 1 diabetes and antitumor response. Yet, several case reports showed improved antitumor response in new-onset type 1 diabetes following PD-1 inhibitors (18). In the present study, all patients showed a good response to their ICI therapy, from stable disease to even complete remission. Even three patients who discontinued the treatment remain stable disease without further chemotherapy for a while. Our findings may provide additional evidence of improving antitumor responses in patients who developed autoimmune diabetes after ICI therapy. However, it should be noted that according to current guidelines, the ICI treatment should be temporarily interrupted and not withdrawn permanently at the onset of DKA.

In summary, we report four Korean cases of ICI-induced DKA, three cases with new-onset type 1 diabetes and one case with known type 2 diabetes. The PD-1/PD-L1 blockades were the major ICIs related to autoimmune diabetes. The presence of autoantibodies against β-cell was observed in one patient (25%). Since there are no practically available predictive biomarkers for the occurrence of DKA or type 1 diabetes following ICI therapy, close monitoring of blood glucose levels should be considered in all patients receiving ICI therapy regardless of the previous history of diabetes.

## Ethics Statement

This study was approved by the Institutional Review Board of Chonnam National University Hwasun Hospital and was conducted in accordance with the Declaration of Helsinki. Retrospective written informed consent was obtained from the participants for the publication of this case report.

## Author Contributions

AH and H-CK managed the case. AH drafted the manuscript. AH, JY, HK, and H-CK reviewed the manuscript. All authors agreed with the final version of the manuscript.

### Conflict of Interest

The authors declare that the research was conducted in the absence of any commercial or financial relationships that could be construed as a potential conflict of interest.
